# Real-world patient’s practices in the management of allergic rhinitis in the Philippine setting

**DOI:** 10.5415/apallergy.0000000000000214

**Published:** 2025-07-11

**Authors:** Ma. Lourdes B. Enecilla, Marysia Stella T. Recto, Cecilia Gretchen Navarro-Locsin, Joel A. Romualdez, Mary Anne R. Castor, Victoria Chato-Andeza, Antonio Hao Chua, Eloisa S. De Guia, Charito C. de los Santos, Heidilita M. Espinoza, Jenifer R. Otadoy-Agustin, Josephine B. Ramos, Ma. Fredelita G. Carreon-Asuncion, Jean Bousquet

**Affiliations:** 1 Department of Otorhinolaryngology – Head and Neck Surgery, St. Luke’s Medical Center, Taguig, Philippines; 2 Division of Allergy and Immunology, University of the Philippines Manila - Philippine General Hospital, Manila, Philippines; 3 Department of Otorhinolaryngology – Head and Neck Surgery, St. Luke’s Medical Center, Quezon City, Philippines; 4 Department of Internal Medicine, Manila Doctors Hospital, Manila, Philippines; 5 Department of Otorhinolaryngology – Head and Neck Surgery, University of the East – Ramon Magsaysay Memorial Medical Center, Quezon City, Philippines; 6 Department of Internal Medicine, Veterans Memorial Medical Center, Quezon City, Philippines; 7 Division of Pulmonary Medicine and Critical Care, Section of Pediatric Pulmonology, Philippine Heart Center, Quezon City, Philippines; 8 Department of Pediatrics, Dr. Jesus C. Delgado Memorial Hospital, Quezon City, Philippines; 9 Section of Pulmonary Medicine, The Medical City- Ortigas, Pasig, Philippines; 10 Program of Internal Medicine, San Beda University College of Medicine, Manila, Philippines; 11 Inserm, CESP Centre for Research in Epidemiology and Population Health, U1018, Respiratory and Environmental Epidemiology team, Villejuif, France

**Keywords:** Adult, allergic rhinitis, child, disease management, health seeking behavior, Philippines

## Abstract

**Background:**

Allergic rhinitis is a prevalent disease and there is a need for local real-world data to create relevant guidelines and care pathways in its management.

**Objective:**

The aim was to investigate the health-seeking behavior and practices of Filipinos in managing allergic rhinitis symptoms.

**Methods:**

A cross-sectional stratified online survey was conducted among Filipinos with allergic rhinitis symptoms. Stratified sampling, based on age group and region of residence in the Philippines, with proportional allocation, was used to select the respondents of this study.

**Results:**

A total of 317 respondents (213 adults and 104 pediatric) were included in the analysis. The majority (61.83%) had moderate-severe intermittent allergic rhinitis. Eighteen percent (18%) had no prior consult with a physician, while 37% had self-medicated. Sixty-three percent of participants preferred taking prescribed oral medications and 48.6% preferred a prescribed nasal spray. The most common drug classes used for allergic rhinitis were oral antihistamines (68.14%) and steroid nasal spray (34.07%). Only 45.42% admitted to being fully adherent with their prescribed medications. The most frequently cited factors that would improve compliance were cost (47.32%), few side effects (47.32%), and rapid onset of effect (40.38%). Only 21.14% of respondents had undergone allergen skin testing and 6.62% had undergone immunotherapy.

**Conclusions:**

Many Filipinos who suffer from allergic rhinitis symptoms have not been seen by a physician or, even after consultation, are not adherent to prescribed medications. There is a need for increased public awareness regarding allergic rhinitis and more effective communication between patients and the healthcare provider to improve outcomes.

## 1. Introduction

In the Philippines, allergic rhinitis is a significant health concern affecting 20% of adult Filipinos based on the National Nutrition and Health Survey (NNH) in 2008 [1]. In children, the 1997 International Study of Asthma and Allergies in Childhood (ISAAC) reported a prevalence of 9.2% in 6 to 7-year-olds and 15.3% in 13 to 14-year-olds [2]. Filipino patients report nasal congestion as the most disturbing symptom and sleep to be the most affected daily activity [3].

The use of evidence-based guidelines affords better allergic rhinitis symptom control. In the Philippines, more than 8 out of 10 specialists in Metro Manila self-report adherence to the Allergic Rhinitis and Its Impact on Asthma (ARIA) guideline [4]. The 2020 ARIA guideline reports that the selection of allergic rhinitis pharmacotherapy depends on (1) patient empowerment, preferences and age; (2) prominent symptoms, symptom severity and multimorbidity; (3) efficacy and safety of treatment; (4) speed of onset of action of treatment; (5) current treatment; (6) historic response to treatment; (7) effect on sleep and work productivity; (8) self-management strategies and (9) resource use [5]. Awareness of these varied factors that can affect a patient’s compliance with pharmacotherapy can help the physician in choosing the most appropriate treatment for the patient.

Successful pharmacotherapeutic management of allergic rhinitis is affected by patient preference and self-management strategies. In a large-scale online survey in the USA, 39% of adults only took medications prescribed by either a physician or a pharmacist while 34% self-medicated with over-the-counter (OTC) medications. Adherence to medications was low [6]. In a local survey on Filipinos residing in the National Capital Region with symptoms of allergic rhinitis, the majority (92%) self-medicated for their symptoms. Among those who used prescribed medications, 92% reported adherence to the drug dosage and duration of therapy [3].

Currently, there is limited local real-world evidence on Filipinos’ health-seeking behavior and practices in addressing their allergic rhinitis symptoms, consultation preferences, self-medication practices, treatment preferences, compliance to prescribed medications, as well as the prevalence of allergen skin testing and immunotherapy.

The current study aimed to gather behavior and practices to create locally relevant guidelines and care pathways in the management of allergic rhinitis.

## 2. Methods

### 2.1. Study design

This was a cross-sectional stratified online survey of Filipinos with allergic rhinitis conducted from July to September 2022.

### 2.2. Ethical compliance

This study was conducted in compliance with the 1964 Helsinki Declaration and its later amendments, and the ethical standards of the institutional and national research committees. Ethical approval for this study (QMMC REB GCS 2022-32) was provided by the Institutional Scientific and Ethical Review Board of Quirino Memorial Medical Center, Quezon City, Philippines (Vice Chair Dr. Rosario Baes) on July 1, 2022.

### 2.3. Setting

Respondents were voluntarily recruited through social media, where they accessed a link to the research study’s social media page. The survey was administered via Google Forms, which was distributed through short message service and other messaging platforms. Informed consent was obtained through Google Forms before proceeding to the survey questions.

### 2.4. Subjects

Respondents were Filipino residents from different regions of the Philippines, aged 2 years and older, who were either previously diagnosed with allergic rhinitis by a physician or presumed to have allergic rhinitis based on their responses to a validated allergic rhinitis questionnaire. Parents or guardians of Filipinos with allergic rhinitis aged between 2–18 y/o were asked to answer the online survey on behalf of the child. In this study, allergic rhinitis was defined as the presence of at least 1 recurring nasal symptom (ie, sneezing, rhinorrhea, nasal congestion, nasal itchiness) lasting more than 1 hour, not associated with fever, and triggered by environmental factors. Allergic rhinitis duration and severity were classified according to the ARIA 2008 guideline.

### 2.5. Questionnaire

The survey incorporated questions from validated allergic rhinitis questionnaires used in the ISAAC survey, the 2008 NNH Survey, and a published online survey by Meltzer et al. [1, 2, 6]. The questions assessed the respondents’ severity and frequency of allergic rhinitis symptoms, perception of triggers, history of allergen skin testing and/or immunotherapy, consultation preferences, self-medication practices, pharmacotherapy preferences, compliance with prescribed medications, and perceived factors that could improve compliance. The full questionnaire is presented in the Supplement https://links.lww.com/PA9/A64.

### 2.6. Sampling and sample size

The target minimum sample size was 315 respondents (213 adults and 102 pediatric) based on the following information: (1) the prevalence of allergic rhinitis among Filipino adults is 20% [1]; (2) the prevalence of allergic rhinitis among Filipino children is 15% [2]; (3) expected compliance to prescribed allergic rhinitis medications is 92% [3]; and (4) precision set at 3% with confidence level at 95%. The sample size was adjusted using a finite population correction factor for an estimated target population size of 20,141,654 Filipinos with allergic rhinitis.

Responses were collected over 3 months with a final total of 782 respondents (614 adults and 168 pediatric), with the majority of the respondents from the Luzon region. Stratified sampling with proportional allocation was then performed according to age group (ie, adult or pediatric) and location of residence (ie, categorized based on the 3 island groups of Luzon, Visayas, or Mindanao), in order to be representative of the population distribution in the Philippines at the time of the survey. Ultimately, only 317 respondents (213 adults and 104 pediatric) were included in this study.

### 2.7. Data analysis

Demographic characteristics of the patients were summarized using descriptive statistics. Categorical variables were summarized as frequencies and percentages, while quantitative variables were described using the mean and standard deviation. Adult and pediatric patients were compared in terms of sociodemographic and clinical variables collected in the study. Mann-Whitney *U* test was used to compare the symptom severity in the 2 patient groups, while the chi-square test was used to compare the 2 patient groups in terms of other variables. Fisher’s exact test was used when the sample size requirement of the chi-square test was not met. A 5% significance level was used for all the hypotheses tested in the study. StataMP version 14 for Mac (StataCorp LLC, USA) was used for data analysis.

## 3. Results

A total of 317 respondents were included in this study – 213 adults and 104 children and adolescents (Table 1). Nearly all respondents (97.79%) reported more than 1 allergic rhinitis symptom, while 88.64% identified at least 1 common aeroallergen as a trigger. Among the respondents, 69% had intermittent allergic rhinitis and 93% had moderate to severe allergic rhinitis. A higher proportion of adult patients reported the following effects of the symptoms compared to pediatric patients: restriction of daily activities (66.2% vs 50%; *P* = 0.006), restriction in school or work participation (52.11% vs 36.54%; *P* = 0.009), perception of symptoms as troublesome (84.51% vs 73.08%; *P* = 0.015). Reported symptom severity was also significantly higher among adult patients compared to pediatric patients (7.02 ± 2.11 vs 5.98 ± 1.98; *P* < 0.001).

**Table 1.** Demographic profile of the allergic rhinitis patients

Characteristics	Sample, n (%)	Adults, n (%)	Pediatric, n (%)	*P*-value
	317 (100%)	213 (67.19%)	104 (32.81%)	
Age, in years				
2–7	39 (12.3%)		39 (37.5%)	
8–12	40 (12.62%)		40 (38.46%)	
13–18	25 (7.89%)		25 (24.04%)	
19–29	58 (18.3%)	58 (27.23%)		
30–39	86 (27.13%)	86 (40.38%)		
40–49	46 (14.51%)	46 (21.6%)		
50–59	18 (5.68%)	18 (8.45%)		
60–69	4 (1.26%)	4 (1.88%)		
70 and above	1 (0.32%)	1 (0.47%)		
Location of residence				0.764
Luzon	182 (57.41%)	125 (58.69%)	57 (54.81%)	
Visayas	60 (18.93%)	40 (18.78%)	20 (19.23%)	
Mindanao	75 (23.66%)	48 (22.54%)	27 (25.96%)	
Highest educational attainment^†^				0.001
Elementary	8 (2.52%)	1 (0.47%)	7 (6.73%)	
High school	28 (8.83%)	16 (7.51%)	12 (11.54%)	
College	182 (57.41%)	128 (60.09%)	54 (51.92%)	
Postgraduate	94 (29.65%)	67 (31.46%)	27 (25.96%)	
Not applicable	5 (1.58%)	1 (0.47%)	4 (3.85%)	
Allergic rhinitis symptoms				
Watery, runny nose	295 (93.06%)	201 (94.37%)	94 (90.38%)	0.190
Sneezing	297 (93.69%)	201 (94.37%)	96 (92.31%)	0.479
Nasal obstruction	260 (82.02%)	181 (84.98%)	79 (75.96%)	0.050*
Itchy nose	283 (89.27%)	196 (92.02%)	87 (83.65%)	0.024*
Watery, red itchy eyes	240 (75.71%)	178 (83.57%)	62 (59.62%)	<0.001***
Allergic rhinitis triggers				
Cockroach	37 (11.67%)	27 (12.68%)	10 (9.52%)	0.426
Dusty places	265 (83.6%)	184 (86.38%)	81 (77.14%)	0.055
Furred animals	119 (37.54%)	78 (36.62%)	41 (39.05%)	0.628
House dust mites	184 (58.04%)	122 (57.28%)	62 (59.05%)	0.692
Mold	120 (37.85%)	80 (37.56%)	40 (38.1%)	0.876
Pollen	139 (43.85%)	97 (45.54%)	42 (40%)	0.385
Change/extreme temperature	18 (5.68%)	14 (6.57%)	4 (3.81%)	0.325
Change in weather	22 (6.94%)	16 (7.51%)	6 (5.71%)	0.567
Food intake	11 (3.47%)	11 (5.16%)	0 (0.00%)	0.019*^‡^
Strong odors/chemicals	21 (6.62%)	18 (8.45%)	3 (2.86%)	0.061
Soap/shampoo/detergent	7 (2.21%)	6 (2.82%)	1 (0.95%)	0.433^‡^
Fumes/smoke	8 (2.52%)	6 (2.82%)	2 (1.9%)	1.000^‡^
Lack of sleep	2 (0.63%)	2 (0.94%)	0 (0.00%)	1.000^‡^
Fibers	1 (0.32%)	1 (0.47%)	0 (0.00%)	1.000^‡^
Water/seawater	1 (0.32%)	1 (0.47%)	0 (0.00%)	1.000^‡^
Ants	2 (0.63%)	2 (0.94%)	0 (0.00%)	1.000^‡^
Powder	1 (0.32%)	1 (0.47%)	0 (0.00%)	1.000^‡^
I do not know	32 (10.09%)	23 (10.8%)	9 (8.57%)	0.552
Allergic rhinitis classification				0.760
Intermittent	219 (69.09%)	149 (69.95%)	70 (67.31)%)	
Persistent	98 (30.91%)	64 (30.05%)	34 (32.69%)	
Allergic rhinitis severity				0.258
Mild	23 (7.26%)	13 (6.1%)	10 (9.62%)	
Moderate-severe	294 (92.74%)	200 (93.9%)	94 (90.38%)	
Allergic rhinitis classification and severity				0.412
Intermittent, mild	23 (7.26%)	13 (6.10%)	10 (9.62%)	
Intermittent, moderate-severe	196 (61.83%)	136 (63.85%)	60 (57.69%)	
Persistent, moderate-severe	98 (30.91%)	64 (30.05%)	34 (32.69%)	
Effect of symptoms on quality of life				
Symptoms disturb your sleep	210 (66.25%)	143 (67.14%)	67 (64.42%)	0.631
Symptoms restrict your daily activities	193 (60.88%)	141 (66.2%)	52 (50%)	0.006**
Symptoms restrict your participation in school or work	149 (47%)	111 (52.11%)	38 (36.54%)	0.009**
Symptoms are troublesome to you	256 (80.76%)	180 (84.51%)	76 (73.08%)	0.015*
Symptom severity, mean (SD)	6.68 (2.12)	7.02 (2.11)	5.98 (1.98)	<0.001***

† For subjects below 18 y/o, this pertains to the educational attainment of the adult answering for the child.

‡ Fisher’s exact test was performed since the sample size requirement of the chi-square test was not met.

* <0.05,

** <0.01,

*** <0.001.

Two-thirds of respondents experienced symptoms all year round. Among those who experienced symptoms only during certain months, symptoms were most common in June (43.02%), December (43.02%), and July (41.86%).

The most commonly consulted healthcare professionals by all respondents with allergic rhinitis were ears-nose-throat (ENT) specialists/otolaryngologists (33.12%), pediatricians (28.08%), and allergologists (27.44%). Among the pediatric respondents, the majority (67.31%) consulted with their pediatricians. Meanwhile, 18 percent (18%) of the respondents (20.66% adults, 13.46% pediatric) had not seen any physician for their allergic rhinitis symptoms at the time of the survey.

**Figure 1.**
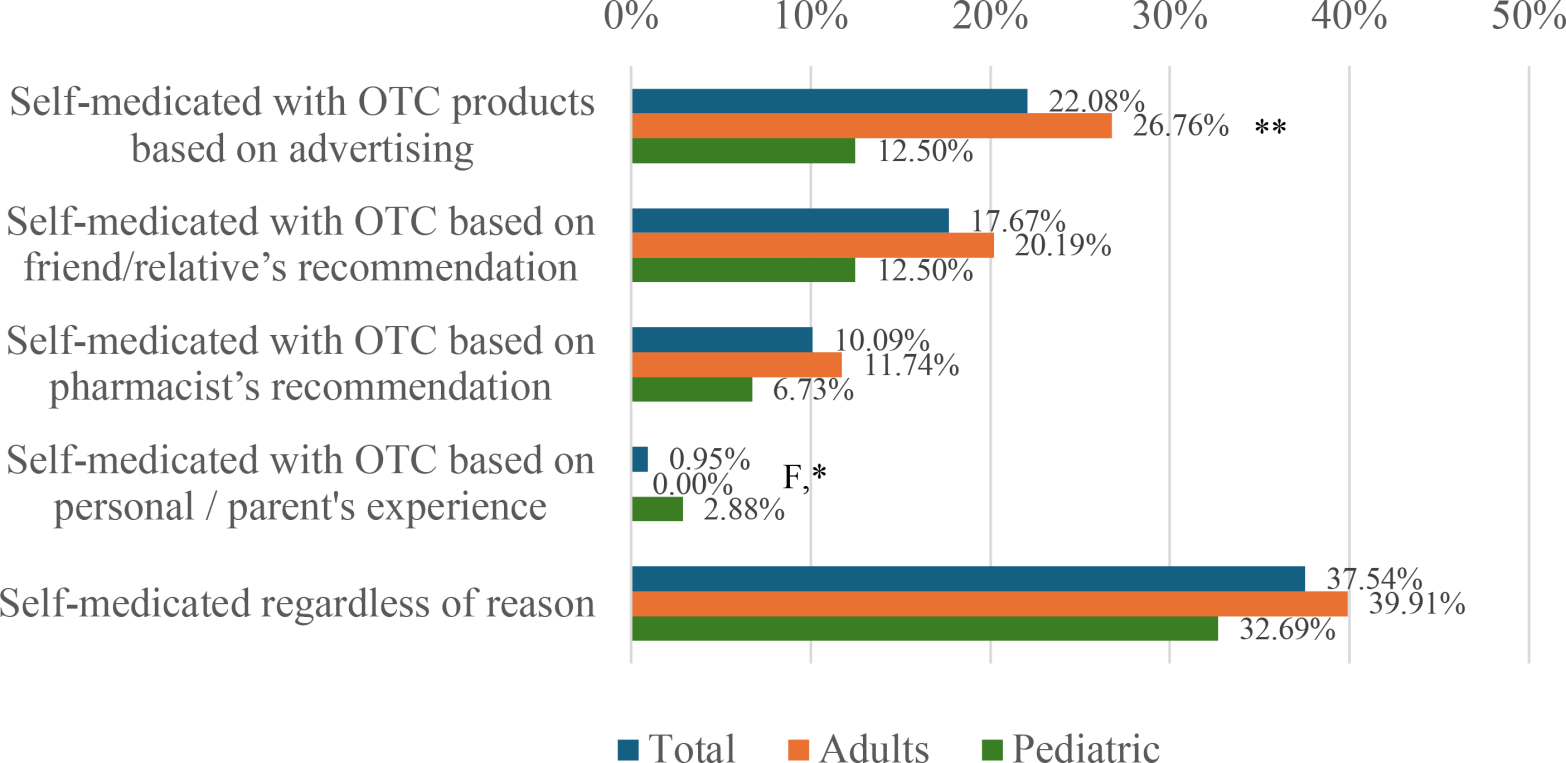
Self-management practices for control of allergic rhinitis symptoms prior to consultation with a physician. F, Fisher’s exact test was performed since the sample size requirement of the chi-square test was not met. *P < 0.05, **P < 0.01.

Figure 1 illustrates how the respondents managed their allergic rhinitis symptoms prior to consultation with a physician. Thirty-seven percent (37%) of respondents self-medicated before consulting with a physician, with most basing their choice on advertising.

**Figure 2.**
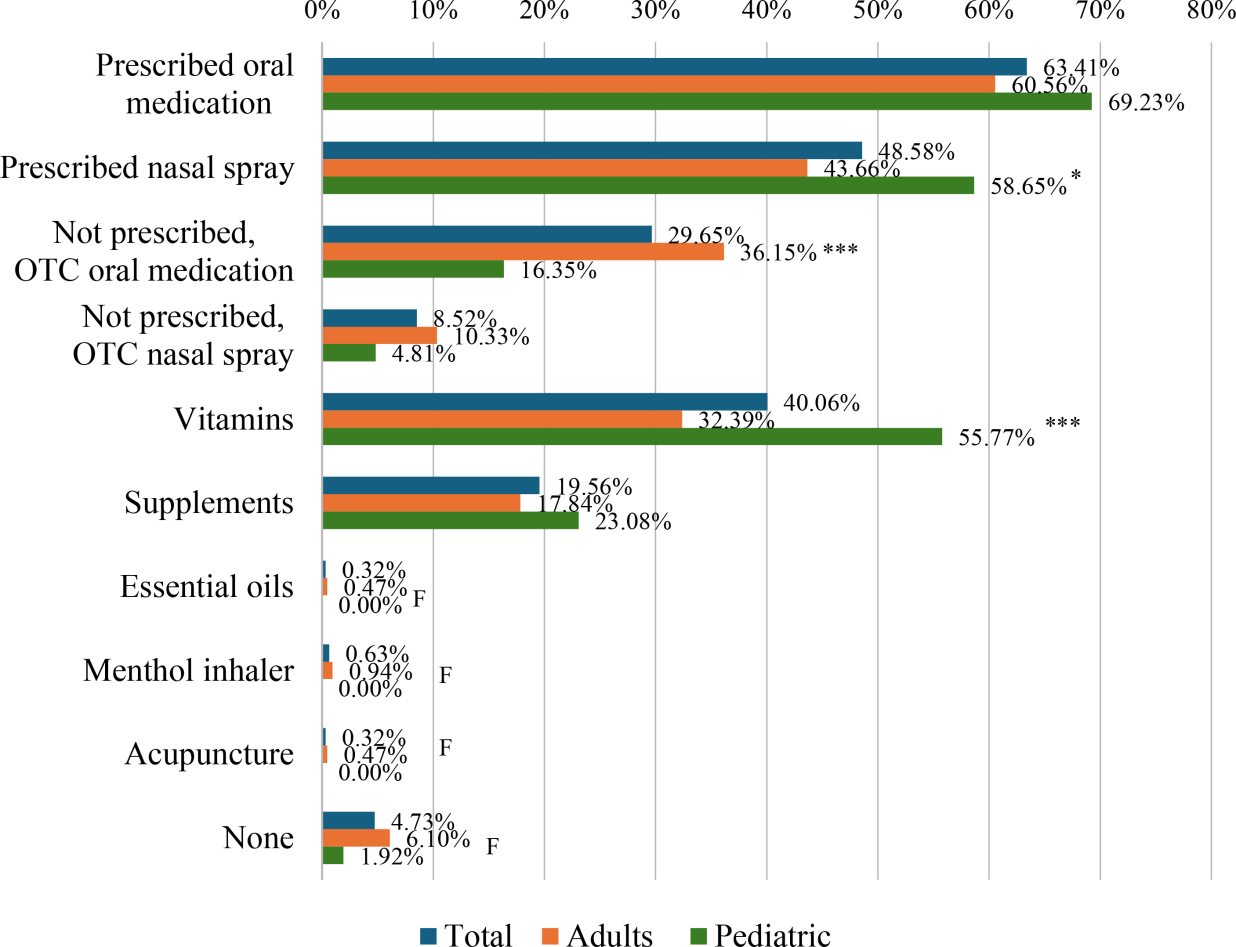
Patients’ pharmacotherapeutic preferences in managing their allergic rhinitis symptoms. F, Fisher’s exact test was performed since the sample size requirement of the chi-square test was not met. *P < 0.05, **P < 0.01, ***P < 0.001.

Figure 2 displays the respondents’ pharmacotherapeutic preferences. In general, the most common pharmacotherapeutic preferences were prescribed oral medications (63.41%), prescribed nasal spray (48.58%), and vitamins (40.06%). A significantly higher proportion of adults preferred OTC oral medications compared to the pediatric respondents (36.15% vs 16.35%; *P* < 0.001). In contrast, pediatric respondents were significantly more likely than adults to receive prescribed nasal sprays (58.65% vs 43.66%; *P* = 0.012) and vitamins (55.77% vs 32.39%; *P* < 0.001).

**Figure 3.**
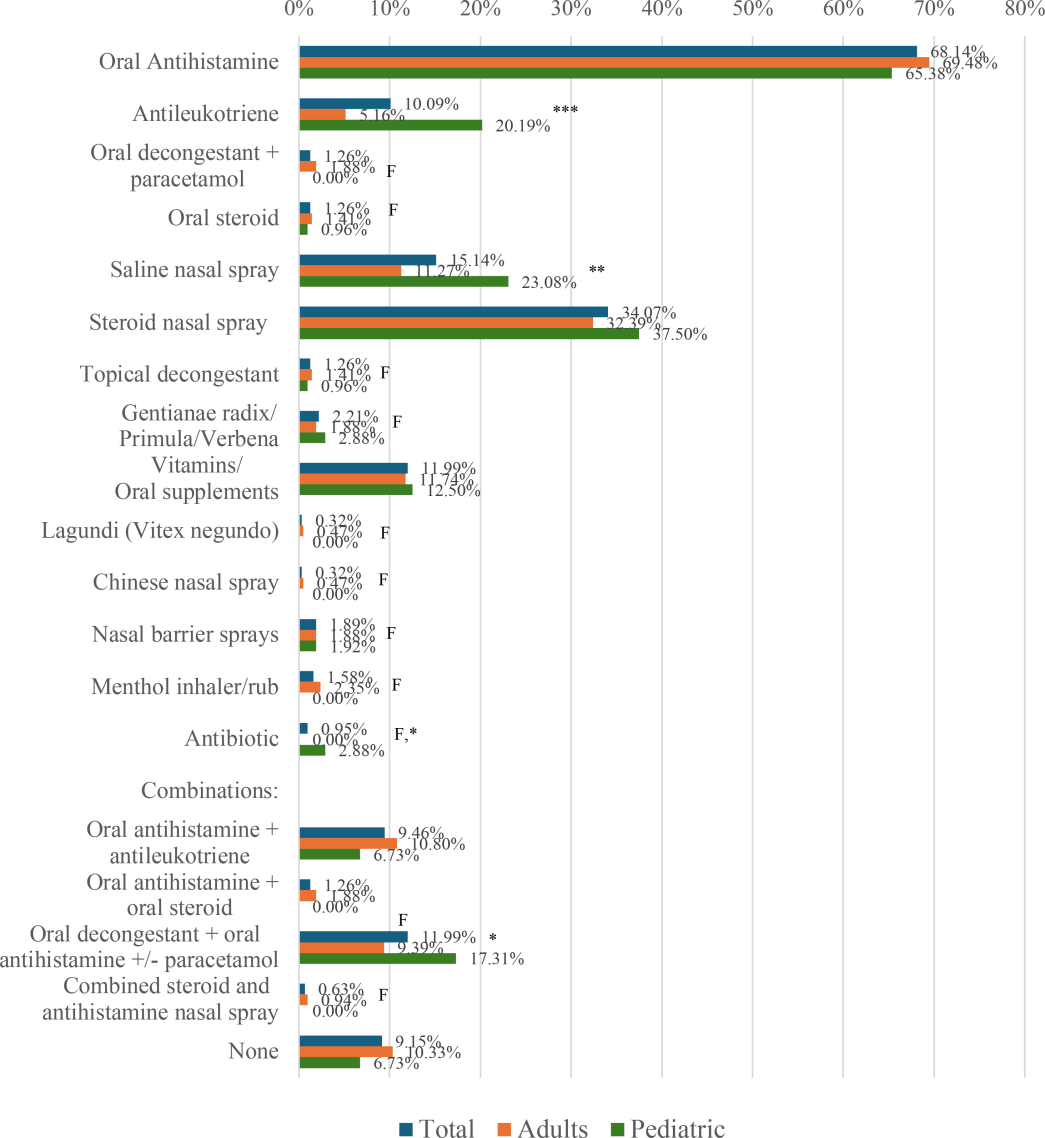
Drug classes used by respondents for managing their allergic rhinitis symptoms. F, Fisher’s exact test was performed since the sample size requirement of the chi-square test was not met. *P < 0.05, **P < 0.01, ***P < 0.001.

Figure 3 presents the classes of drugs respondents used to manage their allergic rhinitis symptoms. The most common classes of drugs used were oral antihistamines (68.14%), intranasal corticosteroids (INCS) (34.07%), and saline nasal spray (15.14%). A significantly higher proportion of pediatric respondents compared to adults used antileukotrienes (20.19% pediatric vs 5.16% adults; *P* < 0.001) and saline nasal spray (23.08% vs 11.27%; *P* = 0.006). Among all respondents, 19% used either antileukotrienes or a combination of antileukotrienes with oral antihistamines (14.55% adults; 26.92% pediatric). For moderate-severe intermittent allergic rhinitis, 31.12% used INCS (30.88% adults; 31.67% pediatric) whereas for moderate-severe persistent allergic rhinitis, 37.76% used INCS (31.25% adults; 50% pediatric).

**Figure 4.**
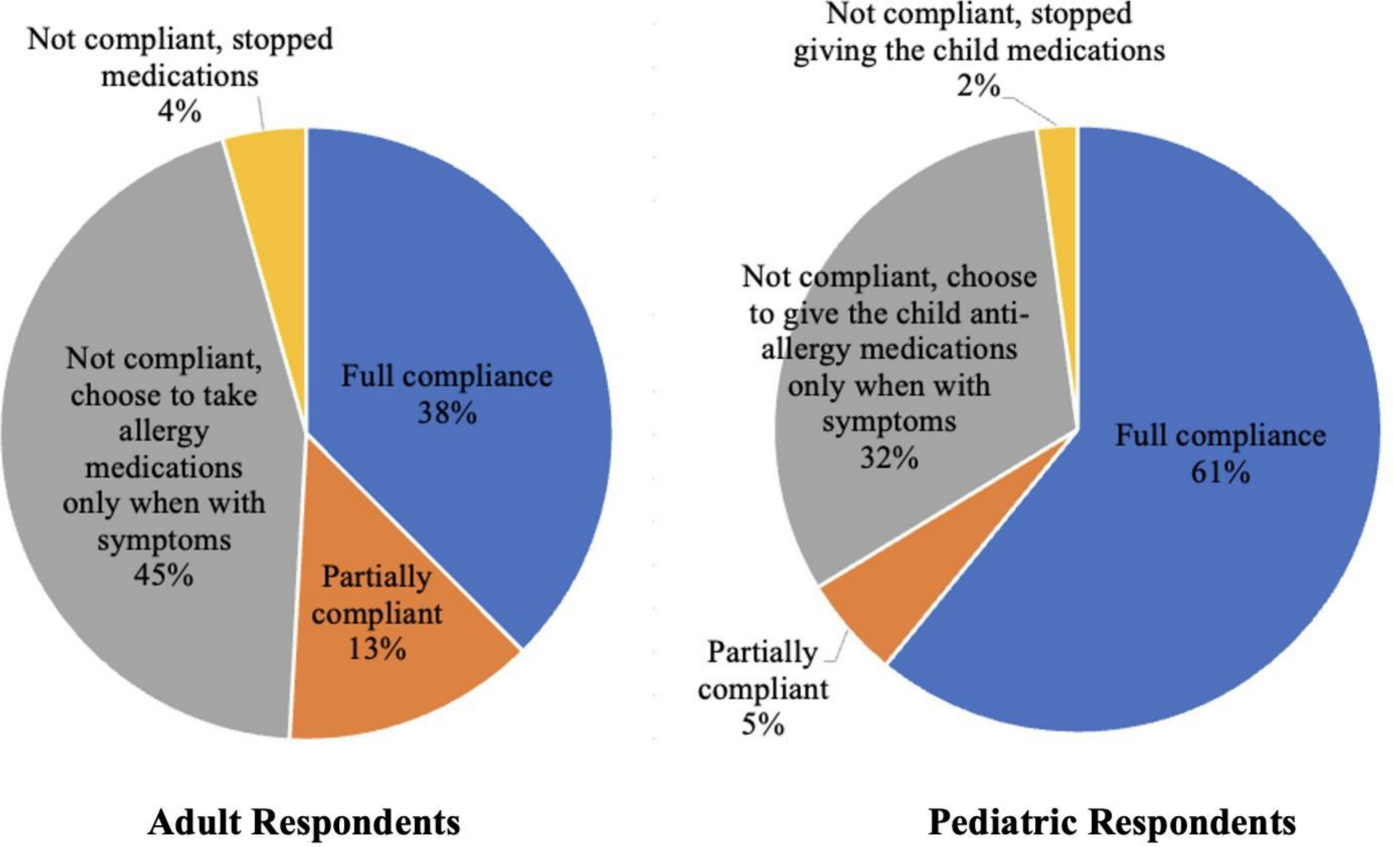
Compliance to prescribed medications for allergic rhinitis.

Figure 4 shows the respondents’ compliance with prescribed medications for allergic rhinitis. Overall, 45.42% of the respondents were fully compliant with their prescribed medications (adults: 37.57%, pediatric: 60.87%).

**Figure 5.**
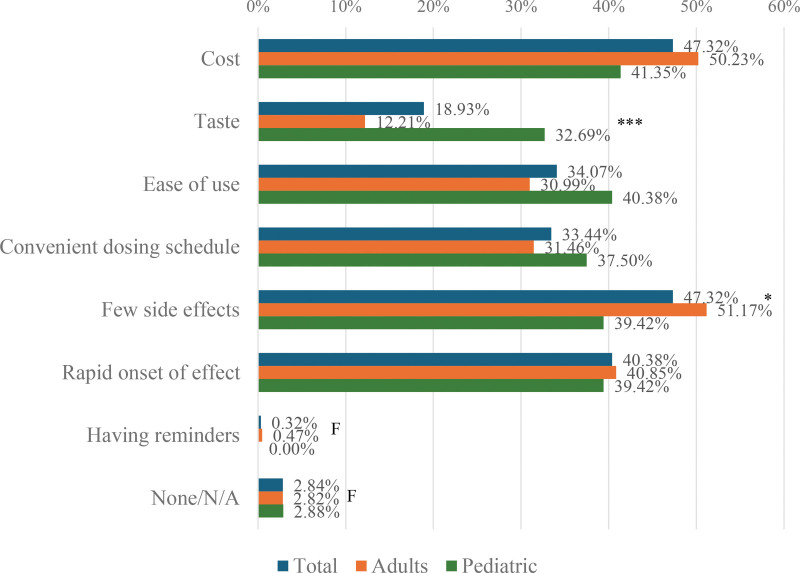
Factors that would improve compliance to prescribed allergic rhinitis medications. F, Fisher’s exact test was performed since the sample size requirement of the chi-square test was not met. *P < 0.05, **P < 0.01, ***P < 0.001.

When asked what factors would improve compliance with prescribed medications, the most frequently cited were cost (47.32%), fewer side effects (47.32%), rapid onset of effect (40.38%), and taste (adults: 32.69%, children: 12.21%; *P* < 0.001), as illustrated in Fig. 5.

Respondents were also asked if they had undergone allergen skin testing and/or immunotherapy. Only 21.14% (95% confidence interval [CI]: 16.62%, 25.65%) of respondents had undergone allergen skin testing and 6.62% (95% CI: 3.87%, 9.38%) had received immunotherapy.

## 4. Discussion

The Philippines is an archipelago consisting of 7,641 islands which are grouped under the 3 major island groups, namely Luzon, Visayas, and Mindanao, and a projected population in 2022 of 111,572,254 [7]. Luzon has the highest population, followed by Mindanao, then Visayas. The majority of previously published studies involving allergic rhinitis were conducted in Luzon [3, 4, 8–10], whereas the last nationwide survey on the prevalence of allergic rhinitis was in 2008 [1]. This current study attempted to get responses from the 3 major island groups in a proportion representative of the entire Filipino population.

All respondents included in the study had at least 1 recurring symptom of allergic rhinitis (ie, sneezing, rhinorrhea, nasal obstruction, nasal itchiness) associated with exposure to environmental triggers and/or were previously diagnosed by a physician to have allergic rhinitis. If the ARIA definition for allergic rhinitis was used, wherein at least 2 recurring symptoms of allergic rhinitis were present, 98% of adult respondents and 97% of pediatric respondents would still fulfill the criteria. Interestingly, 6 out of 7 respondents with only 1 reported symptom associated with allergic rhinitis were previously diagnosed by a physician to have allergic rhinitis. This could mean that the patient’s allergic rhinitis was not being triggered at the time of the survey or that the physician had diagnosed the patient without strictly following the ARIA definition. The most commonly reported triggers for respondents’ symptoms were dusty places, house dust mites, and pollen. In a study by Navarro-Locsin on adult Filipinos with symptoms of allergic rhinitis and at least 1 positive skin prick test (SPT), the most common allergens were *Dermatophagoides pteronyssinus* (97.4%), *Dermatophagoides farinae* (95.8%), and cockroach (80.1%) [8]. In this study, the preponderance of responses indicating “dusty places” as the trigger could indicate a lack of understanding by most Filipinos on what was present in those dusty places that triggered their allergic rhinitis symptoms. Eleven percent (11%) of the respondents either did not know their trigger or reported nonaeroallergens as triggers. Interestingly, 55.56% of these respondents were previously diagnosed by a physician to have allergic rhinitis. This could imply a misdiagnosis, the possible presence of nonallergic rhinitis or mixed rhinitis, and/or a lack of discussion with the patient regarding potential allergens.

In terms of the frequency of allergic rhinitis symptoms, although the results showed that the majority reported having symptoms all year round, when the reported frequency and severity of symptoms were classified using ARIA, the majority were classified as having moderate-severe intermittent allergic rhinitis. The results of this study were similar to those obtained from a large-scale cross-sectional study in Singapore and Malaysia on adult allergic rhinitis patients with positive SPT, wherein only 25.9% had persistent allergic rhinitis based on the ARIA classification [11], and another study involving Australian children wherein the majority of treated allergic rhinitis cases were classified as having moderate-severe, intermittent allergic rhinitis (71.2%) [12]. It is therefore recognized here that having symptoms all year round is not synonymous with the persistent type of allergic rhinitis. In subtropical countries like the Philippines, with long pollinating seasons and the presence of mold and dust mite allergens everywhere, it can be expected that symptoms will occur year-round. However, an individual’s exposure and/or sensitivity may not lead to clinical manifestations on a daily or weekly basis.

In this study, the most frequently consulted physicians by the adult respondents were ENT/otolaryngologists while pediatricians managed the majority of respondents below 18 years old. A previous local cross-sectional survey among physicians found that general physicians see 1 in 10 allergic rhinitis patients per week while specialist physicians (ie, otolaryngologists, pulmonologists, and allergologists) see 1 in 3 allergic rhinitis patients per week [4]. This was most likely due to the current health system in the Philippines wherein patients have the option to consult directly with a specialist, even without being seen first by a generalist.

Although the majority of the respondents consulted a physician for their allergic rhinitis symptoms, 37.54% still self-medicated before consulting with a physician. This is unsurprising since all antiallergy medications in the Philippines can be bought without a prescription. In contrast, a 2016 study by Navarro-Locsin found that 91% to 93% of Filipino allergic rhinitis patients self-medicate [3]. Results from our study may indicate that fewer patients are self-medicating for control of allergic rhinitis symptoms. However, this may also be due to the possibility that the current study caught more of the middle and upper classes by virtue of being an online survey, and possibly more educated respondents who chose to consult a physician instead of self-medicating. Among those who self-medicated, the majority based their decision on advertising, and one-third relied on the pharmacist’s recommendation. This proportion was even higher than in a 2017 USA study by Meltzer et al., where only 4% consulted a pharmacist [6]. In comparison, a local study conducted in Cebu, one of the major cities in the Philippines, found that 50% of respondents relied on the pharmacist’s recommendation [13]. This underscores the need for pharmacists to be involved in allergic rhinitis clinical guidelines and patient care pathways.

It was reassuring that the most common pharmacotherapeutic preferences of both adult and pediatric respondents for allergic rhinitis control were prescribed oral medications and nasal sprays. Almost all the respondents used multiple medications to control their allergic rhinitis symptoms. A significantly smaller proportion of the pediatric respondents used nonprescription oral medications compared to the adult respondents, which implied that more caution was practiced in the management of allergic rhinitis among the young. However, it appeared in this survey that it was still a common practice to address allergic rhinitis symptoms with vitamin intake, particularly in the pediatric population.

Similar to the results of other patient surveys, oral antihistamines were the most used drug class for the control of allergic rhinitis symptoms, regardless of disease severity [6]. In this study, only one-third of those with moderate-severe intermittent allergic rhinitis and moderate-severe persistent allergic rhinitis (31.12% and 37.76%, respectively) used INCS despite globally accepted recommendations that this should be the first-line treatment. In a recent study by Balotro-Torres et al. on physician practices in the management of allergic rhinitis in the Philippines, 68.1% of physician respondents reported having prescribed INCS for persistent allergic rhinitis [14]. The reason for this discrepancy could be that this current survey caught respondents who were being managed differently by their physicians, or it was the patient’s preference. In this survey, it seems that the treatment of pediatric patients with allergic rhinitis adheres more to current guidelines, as 50% of pediatric respondents with moderate-severe persistent allergic rhinitis were on INCS. This may also correlate with the better compliance seen among pediatric respondents compared to adults. This survey also showed that 10% of all respondents used antileukotrienes to treat allergic rhinitis, with a significantly higher proportion among pediatric respondents. The preferential use of antileukotrienes in the local setting for the pediatric population could be due to their better palatability and ease of administration. This practice was corroborated by the study of Navarro-Locsin on physician prescribing practices wherein combination therapy using an antihistamine with antileukotriene, and combination antihistamine with antileukotriene plus INCS were the top 2 combination therapies for moderate to severe allergic rhinitis [4]. In the more recent local study by Balotro-Torres et al, 33.5% of physician respondents prescribed a combination oral antihistamine with antileukotrienes for intermittent allergic rhinitis, whereas for persistent allergic rhinitis, 47.6% prescribed a combination oral antihistamine with antileukotrienes, and 29% prescribed antileukotrienes alone [14]. However, as early as 2018, the International Consensus Statement on Allergy and Rhinology (ICAR) recommended against the use of antileukotrienes as first-line therapy for allergic rhinitis but may be used as second-line therapy (eg, when INCS are contraindicated) or as add-on treatment [15], a recommendation reiterated in the 2023 ICAR [16]. In the United States, Montelukast, an antileukotriene, carries a black-box warning regarding possible neuropsychiatric side effects including suicidal ideation. No such warning is prominently shown in the locally distributed products. This again underscores the need for local clinical practice guidelines and care pathways, to guide local physicians and pharmacists. It would also be useful to conduct local studies on the occurrence of neuropsychiatric side effects among Filipinos using Montelukast. It should also be noted that several respondents took medications for allergic rhinitis symptoms that are not part of the accepted allergic rhinitis management guidelines such as antibiotics, menthol inhalers, nasal barrier sprays, and phytomedicines such as the internationally-available combination secretolytic drug Gentianae radix/Primula flos/Verbenae herba/Rumicis herba/Sambuci flos and the locally available antitussive and bronchodilator Lagundi *(Vitex negundo*) [17]. This highlights the need for better education campaigns regarding the appropriate medications for allergic rhinitis for although most of the medications are relatively safe, this still adds to the unnecessary expenditure by the patient. It also reinforces the important role of pharmacists in advising patients on the use of OTC drugs.

Compliance with prescribed medications is another important factor in allergic rhinitis management. In this study, only 39.12% of the patients were fully compliant with their medications. To disaggregate, compliance among adults was 31.92%, which was significantly lower than the 53.85% compliance observed among pediatric patients. Interestingly, a greater proportion (45%) of the adult respondents who had consulted with a physician chose to take the prescribed medications only when symptomatic, which may explain the preference for medications with a fast onset of action, such as oral antihistamines.

In this study, compliance with medications was affected by cost, side effects, and onset of drug action. Cost is a major factor in the Philippines wherein despite the creation of a Universal Health Care Program in 2019 and the presence of a National Health Insurance Program, in 2022, 44.2% of healthcare expenditures were from household out-of-pocket payments [18]. Compliance may also be linked to the perception of the severity and impact of allergic rhinitis on the patients’ lives, as well as the perceived dangers of using the prescribed allergic rhinitis treatment. Hence, better patient education on the disease process and rationale for management strategies must be included during physician consultation. Further studies may also be conducted to correlate the perceived severity of patients’ symptoms and compliance. The use of symptom diaries, whether manual or digital, may help tailor the treatment to the patient’s symptoms and improve compliance.

Regarding allergen testing, only 21.14% of the respondents underwent allergen skin tests. Although almost 93% of the respondents had moderate-to-severe allergic rhinitis, only 6.62% had received allergen immunotherapy. Allergen SPTs and immunotherapy are performed by allergologists, and since only 27% of respondents were seen by them, lack of access and awareness may be a contributing factor. Another survey on the knowledge, perceptions, and expectations of Filipino allergic rhinitis patients regarding allergen testing and specific allergen immunotherapy might shed light on why only a few patients have undergone these procedures.

### 4.1. Study limitations

This study utilized an online survey owing to restrictions in place during the COVID-19 pandemic. Unfortunately, this limited respondents to Filipinos with a cellphone or computer and access to the internet. However, based on the 2020 Philippine Census, 56.1% of households, or more than 50 million Filipinos have Internet access [7], suggesting that the study still captured a good representation of the population. The authors also recognize the limitation in the study that it captured mostly the population with College-level education or higher (87.06%), whereas based on the 2020 Philippine Census only 23.7% of the population has attained college-level education or higher [19].

The study questionnaire was only available in English. Translation to the local dialect was not feasible because there are more than 100 ethnic dialects in the Philippines, of which at least 10 are considered major dialects [20]. Although this could possibly limit the respondents for the study, English is the country’s second official language, and Filipinos usually rank high in English proficiency [21]. Therefore, we believe that language is not a major limitation of this study.

This survey documented the real-world behavior and practices of Filipinos in the management of allergic rhinitis, without physician oversight or guidance. The authors recognize that the study may have included respondents with nonallergic rhinitis. The authors strived to create a survey using validated and unambiguous questions. However, responses still relied heavily on the respondent’s perception of their illness and how they understood the questions. This may contribute to some error or bias in the results but the authors believe that it still gives valuable insight on gaps in knowledge and health education.

## 5. Conclusion

Based on this online survey involving respondents from the 3 major island groups of the Philippines, the majority of respondents had moderate-severe intermittent allergic rhinitis. While the majority of the respondents sought consultation with a physician, commonly with an otolaryngologist, pediatrician, or allergologist, a third self-medicated. The majority of the respondents took oral antihistamines, whether prescribed or not, regardless of disease severity. Less than half of the respondents who sought physician consultation were compliant with their treatment. Consideration of patient risk factors, symptoms, comorbidities, and preferences to tailor-fit the treatment plan may improve outcomes and increase compliance. The use of cost-effective, rapid-onset, and longer-acting therapies with fewer adverse reactions may also improve patient satisfaction. Effective communication between patients and healthcare providers to include information on the disease process, prevention or control strategies such as avoidance of aeroallergens, and different management strategies, including immunotherapy, empowers the patient to be involved in the management of their disease and may improve allergic rhinitis outcomes. This study provides insights into the factors affecting allergic rhinitis treatment perception and compliance, offering valuable data for crafting strategies, guidelines, and policies to improve allergic rhinitis patient outcomes.

## Acknowledgements

Assistance with the study: The authors would like to thank Dr. Maria Bettina Quiambao for the assistance in data collection, Professor Kim L. Cochon for the statistical analysis, and United American Pharmaceuticals for the support.

Presentation: 21st Philippine Society of Allergy, Asthma and Immunology Annual Convention. September 7, 2023. New World Makati Hotel, Philippines.

## Conflicts of interest

The authors have no financial conflicts of interest.

## Author Contributions

All authors collaborated in the conception and design of the study, data collection, and preparation of the manuscript. Ma. Lourdes B. Enecilla served as the primary investigator, coordinated activities, and drafted and finalized the manuscript. Marysia Stella T. Recto initiated and supervised the project, and critically reviewed and edited the manuscript. Cecilia Gretchen Navarro-Locsin, Joel A. Romualdez, and Jean Bousquet critically reviewed and edited the manuscript. All authors read and approved the final manuscript.

## Supplementary material

Supplementary Material can be found via 10.5415/apallergy.2022.12.e38
